# Paraplegia caused by a thoracic angiolipoma obscured by cord injury: a case report

**DOI:** 10.3389/fmed.2026.1837857

**Published:** 2026-07-17

**Authors:** Tae Hoon Yang, In-Suk Bae, Hee In Kang, Jae Hoon Kim, Cheolsu Jwa, Eun Kyung Kim

**Affiliations:** 1 Department of Neurosurgery, Nowon Eulji Medical Center, Eulji University, Seoul, Republic of Korea; 2 Department of Pathology, Nowon Eulji Medical Center, Eulji University, Seoul, Republic of Korea

**Keywords:** angiolipoma, cervical cord injury, paraplegia, spinal epidural angiolipomas, tumor

## Abstract

**Background:**

Spinal epidural angiolipomas are rare benign tumors comprising 0.5–1% of all spinal neoplasms and typically arise in the thoracic spine, causing progressive myelopathy due to spinal cord compression. They feature mature adipocytes and vascular proliferation, show heterogeneous magnetic resonance imaging (MRI) enhancement, and may mimic posttraumatic or degenerative myelopathy, particularly in patients with prior spinal cord injury.

**Case description:**

A 56-year-old man presented with a 3-month history of progressive numbness and weakness in both lower limbs. He had sustained a cervical spinal cord injury 5 years earlier and remained ambulatory with a walker after cervical laminoplasty. At initial evaluation, neurological examination demonstrated symmetric lower-extremity weakness, a sensory level below T12, hyperreflexia in both legs, and preserved bladder and bowel function. Cervical MRI showed chronic postoperative change at C5-6 without a new compressive lesion, and the symptoms were initially attributed to sequelae of the prior cervical injury. During 1 month of observation, the patient developed worsening paraparesis and progressive sensory deficit. Whole-spine MRI then revealed a posterior epidural mass at T11-12 causing marked cord compression and extending into the right neural foramen. The lesion showed high signal intensity on T1- and T2-weighted images with heterogeneous contrast enhancement, favoring a vascularized fat-containing tumor. The patient underwent T11-12 laminectomy and gross total resection. Histopathological examination confirmed epidural angiolipoma composed of mature adipocytes and proliferating vascular channels. Postoperatively, neurological symptoms improved without complication, and the patient remained improved at 1-month follow-up.

**Conclusion:**

Spinal epidural angiolipoma should be considered in patients with prior spinal cord injury who present with new-onset myelopathy. Whole-spine MRI screening is critical for preventing diagnostic delays and facilitating favorable surgical outcomes.

## 1 Introduction

Spinal epidural angiolipomas are rare benign tumors composed of mature adipose tissue interspersed with proliferating vascular elements (1, 2). They account for approximately 0.04–1.2% of all spinal tumors and represent 2–3% of tumors arising in the epidural space (1, 3, 4).

Spinal epidural angiolipomas most commonly present with slowly progressive symptoms of spinal cord compression. However, preoperative diagnosis can be challenging because the imaging features are often nonspecific. Although typically asymptomatic until adulthood, these lesions may mimic other conditions, such as degenerative disease or post-traumatic myelopathy, particularly in patients with a history of spinal cord injury, leading to potential diagnostic delay.

Here, we report a rare case of thoracic epidural angiolipoma initially masked by a history of cervical spinal cord injury and emphasizes the importance of correlating the neurological examination with whole-spine imaging.

## 2 Case description

A 56-year-old man presented with a 3-month history of progressive numbness and weakness in both lower limbs. Five years earlier, he had sustained a cervical spinal cord injury (SCI) following a fall. At that time, the patient underwent cervical laminoplasty for decompression of severe cord compression and subsequently remained ambulatory with the aid of a walker.

### 2.1 Clinical findings at initial presentation

At the first evaluation, mental status and cranial nerve examination were unremarkable. Motor examination showed symmetric paraparesis with Medical Research Council grade 4/5 strength in both lower extremities; upper-extremity strength was preserved. Sensory examination demonstrated decreased sensation below the T12 dermatome at the time of initial presentation. Deep tendon reflexes in both lower extremities were increased, and long-tract involvement was clinically suspected. No new bladder or bowel dysfunction was present. Because the patient already had a prior history of cervical spinal cord injury with residual gait impairment, the initial clinical deterioration was interpreted as possible progression of cervical sequelae.

Initial cervical spine MRI demonstrated chronic postoperative changes centered at C5-6 compared with imaging obtained 4 years earlier, but no definite new cervical compressive lesion explaining the newly recognized thoracic sensory level (Figure 1). In retrospect, the presence of a T12 sensory level at the first evaluation should have prompted broader neuraxial investigation earlier.

**Figure 1:**
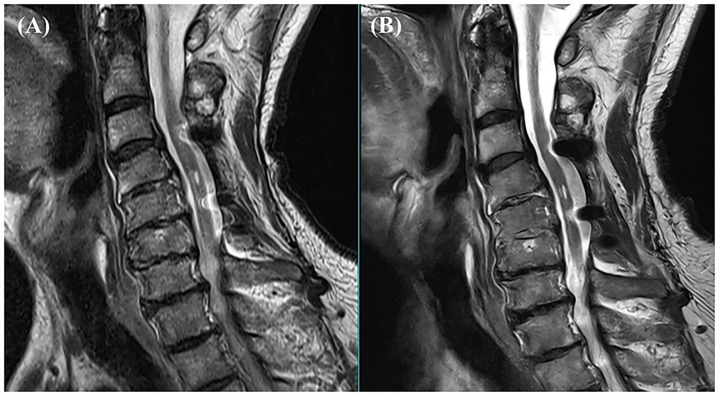
Cervical spine sagittal T2-weighted MRI obtained **(A)** 4 years before presentation and **(B)** at the current presentation, demonstrating chronic postoperative changes following prior cervical decompression without a new lesion explaining the thoracic sensory level.

### 2.2 Clinical course and imaging

The patient was observed for 1 month, during which his neurological status worsened. Lower-extremity motor power declined to grade 3/5 bilaterally, and the sensory deficit became more pronounced. Because the progression was not satisfactorily explained by the cervical findings, whole-spine MRI was obtained.

Thoracic MRI revealed a posterior epidural mass at T11-12 causing marked compression of the thoracic spinal cord. On axial and sagittal images, the lesion was multilobulated and extended into the right T11-12 neural foramen. It showed high signal intensity on both T1- and T2-weighted sequences and marked heterogeneous enhancement after contrast administration, consistent with a vascularized fat-containing epidural tumor (Figure 2). The lesion compressed and displaced the cord anteriorly. No additional compressive lesion was identified elsewhere in the spine.

**Figure 2:**
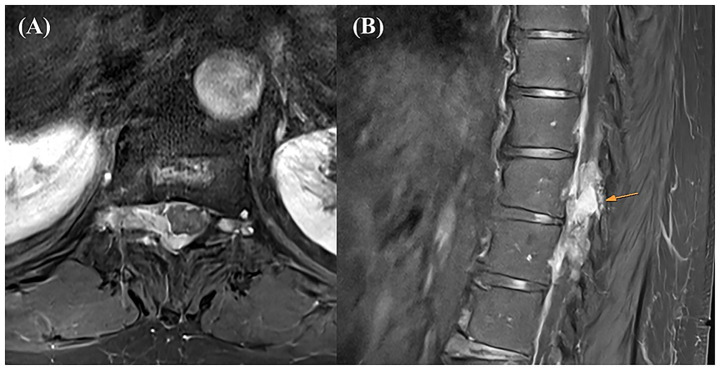
Contrast-enhanced thoracic spine MRI of the epidural angiolipoma at T11-12. **(A)** Axial image and **(B)** sagittal image reveal a posterior multilobulated epidural mass extending into the right T11-12 neural foramen, with high T1- and T2-signal intensity and heterogeneous enhancement, causing marked spinal cord compression.

### 2.3 Surgical treatment and pathology

The patient underwent T11-12 posterior decompression through subtotal laminectomy with microsurgical gross total resection of the epidural mass. The lesion was located in the posterior epidural space and was removed without intraoperative complication. Adequate decompression of the thecal sac was achieved, and no dural violation or postoperative cerebrospinal fluid leakage occurred.

Histopathological examination demonstrated a well-circumscribed lesion composed of mature adipose tissue admixed with numerous thin-walled proliferating vascular channels, consistent with epidural angiolipoma. Immunohistochemical staining for CD34 highlighted the vascular component (Figure 3).

**Figure 3:**
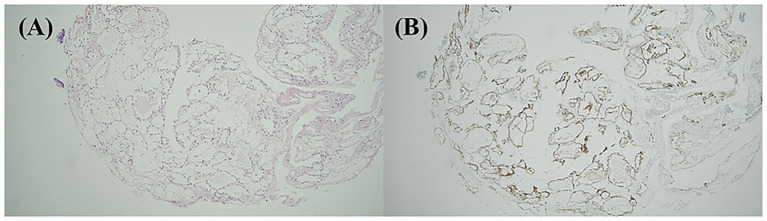
Histopathological examination of the tumor. **(A)** Well-circumscribed lesion composed of mature adipose tissue admixed with numerous thin-walled vascular channels (hematoxylin and eosin, ×100). **(B)** Immunohistochemical staining for CD34 highlights endothelial cells lining the vascular structures, confirming the vascular component of the angiolipoma (CD34, ×100).

### 2.4 Follow-up and outcome

After surgery, lower-extremity strength and sensation gradually improved. At 1-month follow-up, the patient had marked neurological improvement compared with the preoperative state and had no surgical complication. Although the follow-up period remains short, the early postoperative course was favorable.

### 2.5 Timeline

- 5 years before presentation: Cervical spinal cord injury after a fall; cervical laminoplasty performed.
- After rehabilitation: Ambulatory with walker.
- 3 months before admission: Progressive bilateral lower-extremity numbness and weakness began.
- Initial evaluation: Paraparesis with sensory level below T12; cervical MRI showed chronic postoperative change only.
- 1 month later: Worsening paraparesis and sensory deficit.
- Follow-up imaging: Whole-spine MRI revealed T11-12 epidural mass with cord compression.
- Surgery: T11-12 subtotal laminectomy and gross total tumor resection.
- 1 month postoperatively: Neurological improvement without complication.

## 3 Discussion

Angiolipomas are common benign tumors that typically develop within the subcutaneous tissues of the forearm, trunk, neck, and proximal upper extremities (5). In contrast, spinal angiolipomas are rare and most frequently occur in the posterior epidural space of the thoracic spine. The term angiolipoma was first introduced by Howard and Helwig in 1960 and is classified into two subtypes: encapsulated and infiltrative. The encapsulated, noninfiltrating type usually demonstrates benign behavior and carries an excellent prognosis, whereas the infiltrative, nonencapsulated variant may display locally aggressive features and less favorable outcomes (6–8). These lesions are distinct from spinal lipomas, which typically occur in the lumbar region and are often associated with spinal dysraphism (9).

Spinal cord dysfunction resulting from such lesions can lead to significant neurological and psychosocial consequences that affect the motor, sensory, and autonomic systems. Although the epidemiology of traumatic paraplegia is well established (10), non-traumatic acute or subacute paraplegia remains poorly documented. Several spinal pathologies, including vascular malformations and tumors associated with intramedullary hemorrhage, can lead to sudden paraplegia without antecedent trauma.

The pathophysiology of spinal cord injury (SCI) comprises two phases: an initial primary phase followed by a prolonged secondary phase that may persist for days to months (11–13). The secondary phase involves mechanisms such as neuroinflammation, ischemia, free radical formation, lipid peroxidation, blood–brain barrier disruption, edema, protease release, and excitotoxicity (13). These processes can exacerbate neurological deficits, resulting in worsening weakness, rigidity, and sensory disturbances. In our case, the patient had a history of cervical SCI with residual lower limb paralysis, which initially led to misinterpretation of the presenting symptoms as secondary deterioration of the prior injury. This focus on the cervical lesion contributed to a delay in recognizing the underlying thoracic pathology.

Typical clinical manifestations of spinal epidural angiolipomas include progressive paraparesis, sensory disturbances in the lower extremities, and hyperreflexia. Acute neurological deterioration may occasionally occur due to intratumoral thrombosis or hemorrhage (14, 15). In such cases, urgent decompression is the treatment of choice to prevent irreversible deficits. Surgical decompression with total tumor resection has been consistently associated with excellent postoperative neurological recovery (9, 15–17). In our case, although diagnosis was somewhat delayed, surgical intervention was undertaken promptly once the diagnosis was established, and the patient achieved a favorable outcome.

Spinal epidural angiolipomas account for less than 1% of all spinal tumors and are rarely reported in patients with a history of SCI. In the present case, the T11–12 lesion mimicked recurrent cervical cord dysfunction, illustrating that atypical myelopathic presentations can obscure the underlying pathology. The principal value of this case lies in its diagnostic message. This patient had a known cervical spinal cord injury, which created a strong potential for diagnostic anchoring. However, the new symptoms were not fully explained by the prior lesion, particularly because a thoracic sensory level was already present at the first evaluation. In retrospect, this finding should have prompted more immediate consideration of a thoracic lesion and earlier whole-spine imaging.

This case also underscores the importance of a complete neurological examination in patients with prior spinal pathology. Careful localization based on motor findings, reflex changes, and a defined sensory level can identify a discordance between symptoms and known imaging abnormalities. When progressive myelopathy is observed and the clinical picture is not fully concordant with a prior lesion, the evaluation should not be limited to the previously injured segment.

The radiologic features in this case were also informative. The T11-12 epidural mass was posterior, multilobulated, extended into the right neural foramen, demonstrated high T1 and T2 signal intensity, and enhanced heterogeneously after contrast administration. Together with the pathology showing mature adipocytes and proliferating vascular channels, these findings supported the diagnosis of spinal epidural angiolipoma.

This report has several limitations. Follow-up was short, the patient perspective could not be obtained for inclusion, and the clinical course was reconstructed retrospectively from the medical record. Nevertheless, the case remains educational because it demonstrates how reliance on a known cervical lesion may delay recognition of a new and surgically treatable thoracic compressive pathology.

## 4 Conclusion

Although spinal epidural angiolipoma is rare, it should be considered in patients with prior spinal cord injury who develop new or progressive myelopathic symptoms. Such symptoms should not automatically be attributed to the known lesion. When examination findings suggest an additional or discordant level of involvement, whole-spine MRI may be necessary to avoid diagnostic delay and facilitate timely surgical treatment.

## Data Availability

The original contributions presented in the study are included in the article/supplementary material, further inquiries can be directed to the corresponding author.
